# Repeated electroacupuncture treatment attenuated hyperalgesia through suppression of spinal glial activation in chronic neuropathic pain rats

**DOI:** 10.1186/s12906-018-2134-8

**Published:** 2018-02-21

**Authors:** Jun-ying Wang, Yong-hui Gao, Li-na Qiao, Jian-liang Zhang, Cheng-Lin Duan-mu, Ya-xia Yan, Shu-ping Chen, Jun-ling Liu

**Affiliations:** 1 Department of Physiology, Institute of Acupuncture and Moxibustion, China Academy of Chinese Medical Sciences, No. 16, Nanxiaojie, Dongzhimennei, Beijing, 100700 China; 2 Department of Biochemistry and Molecular Biology, Institute of Acupuncture and Moxibustion, China Academy of Chinese Medical Sciences, Beijing, 100700 China; 3 Huanghe University of Science and Technology, Henan, 450063 China

**Keywords:** Chronic neuropathic pain, Electroacupuncture, Microgliacytes, Astrocytes, Lumbar spinal cord

## Abstract

**Background:**

Cumulated evidence reveals that glial cells in the spinal cord play an important role in the development of chronic neuropathic pain and are also complicated in the analgesic effect of EA intervention. But the roles of microgliacytes and astrocytes of spinal cord in the process of EA analgesia remain unknown.

**Methods:**

A total of 120 male Wistar rats were used in the present study. The neuropathic pain model was established by chronic constrictive injury (CCI) of the sciatic nerve. The rats were randomly divided into sham group, CCI group, and sham CCI + EA group, and CCI + EA group. EA was applied to bilateral Zusanli (ST36)-Yanlingquan (GB34). The mechanical (both time and force responses) and thermal pain thresholds (PTs) of the bilateral hind-paws were measured. The number of microgliacytes and activity of astrocytes in the dorsal horns (DHs) of lumbar spinal cord (L4–5) were examined by immunofluorescence staining, and the expression of glial fibrillary acidic protein (GFAP) protein was detected by western blot.

**Results:**

Following CCI, both mechanical and thermal PTs of the ipsilateral hind-paw were significantly decreased beginning from the 3rd day after surgery (*P* < 0.05), and the mechanical PT of the contralateral hind-paw was considerably decreased from the 6th day on after surgery (*P* < 0.05). CCI also significantly upregulated the number of Iba-1 labeled microgliacytes and the fluorescence intensity of glial fibrillary acidic protein (GFAP) -labeled astrocyte in the superficial laminae of DHs on bilateral sides (*P* < 0.05). After repeated EA, the mechanical and thermal PTs at bilateral hind-paws were significantly relieved (*P* < 0.05). The increased of number of microgliacytes was markedly suppressed by 2 days’ EA intervention, and the average fluorescence intensity was suppressed by 2 weeks’ EA. The expression of GFAP protein were down-regulated by 1 and 2 weeks’ EA treatment, respectively (*P* < 0.05).

**Conclusions:**

Repeated EA can relieve neuropathic pain and mirror-image pain in chronic neuropathic pain rats, which is probably associated with its effect in downregulating glial cell activation of the lumbar spinal cord, the microgliacyte first and astrocyte later.

## Background

Chronic neuropathic pain, caused by peripheral nerve, is often associated with hyperalgesia, allodynia, and spontaneous pain. It is traditionally considered that the neuronal pain pathway composed of a serial chain of neuronal elements is responsible. However, in recent years, it was found that glial cell activation shown by hypertrophy of astrocytes and ameboid shaping of microgliacytes in the spinal cord following peripheral nerve injury, inflammation, tumor development, etc. also contributes to pain occurrence [1, 2]. The crosstalk among astrocytes, microgliacytes and neurons is closely related with the induction and maintenance of neuropathic pain [3, 4]. Peripheral nerve injury activates microgliacytes and astrocytes, leading to a close contact of their synapses and processes and an aberrant release of many neurotrophic factors and pro-inflammatory cytokines as brain-derived neurotrophic factor (BDNF), glia cell line-derived neurotrophic factor (GDNF), tumor necrosis factor-alpha (TNF-α), interleukin-1 beta (IL-1β), interleukin-6(IL-6), etc. in the dorsal horns (DHs) of the spinal cord and brain, and thereby initiating the pain process [5, 6]. Repeated intrathecal (i.t.) injection of a glial modulating agent propentofylline decreased the mechanical allodynia induced by peripheral nerve injury [7, 8]. Therefore, it is increasingly recognized that the glial activation within the spinal cord DHs is definitely sufficient to induce and maintain pain conditions [9, 10].

Electroacupuncture (EA) intervention is an effective approach for relieving chronic neuropathic pain, and is currently used worldwide due to easy operation and fewer side-effects, but the underlying mechanisms are far more unclear. Previous studies showed that the analgesic effect of EA is mediated by the descending pain inhibitory pathways, also involving spinal opioids, adrenergic, dopaminergic, serotonergic, cholinergic receptors, etc. [11, 12].

In recent years, some reports revealed that glial cells in the spinal cord are also involved in the process of EA analgesia in different pain models. It has been revealed that the analgesic effect of EA may be partly mediated by inhibition of inflammation and glial activation [13]. In monoarthritis rats, multiple EA interventions significantly inhibited spinal glial activation and behavioral hypersensitivity, and when EA plus fluorocitrate (a glial metabolic inhibitor) administrated, the analgesic effect of EA was significantly enhanced [14]. EA combined with low dose of propentofylline produced a stronger anti-allodynia relevant to propentofylline or EA alone [15]. Choi reported that acupuncture stimulation of Shuigou (GV26) and Yanglingquan (GB34) decreased the proportion of the activated microgliacytes, and inhibited both p38 mitogen-activated protein kinase (MAPK) and extracellular signal-regulated kinase (ERK) activation in microgliacytes at the L4–5 [16]. However, it remains largely unknown about the different roles of astrocytes and microgliacytes in the DHs of lumbar spinal cord in the process of EA analgesia.

Our past study [6] demonstrated that repeated EA treatment effectively relieved chronic neuropathic pain and suppressed the elevated expression of IL-1β mRNA and TNFα mRNA, and BDNF, nerve growth factor(NGF), and neurotrophin (NT)3/4 proteins in the spinal cord, some of which have been demonstrated to be involved in spinal cord reactive astrogliosis [17, 18]. But the roles of microgliacytes and astrocytes in EA analgesia at different time-points of chronic constrictive injury (CCI) of sciatic nerve have not been determined. Hence, the present study was designed to investigate the effects of EA on astrocytic and microglial activities in the early induction phase and maintenance phase of neuropathic pain in CCI rats.

## Methods

### Animals

Adult male Wistar rats weighing 200-250 g purchased from the Military Academy of Medical Sciences [Animal Certificate No.: SCXK (Army) 2012–0004] (Beijing, the People’s Republic of China). Animals housed in 540X395X200 mm^**3**^ cages (Suzhou Qiaofeng Experimental Animal Cage Factory, China, RS-R5) were acclimatized to standard laboratory conditions (light-controlled about 12:12 h light-dark cycles) and given free access to standard chow pellet diet and water. During housing, the animals were monitored twice daily for health status, and handled for about 10 min in the morning, once daily, continuously for at least 3 days for reducing stress responses. All procedures were performed in accordance with the “*Guidelines for Laboratory Animal Care and Use*” from the Chinese Ministry of Science and Technology (2006) and approved by the Institute of Acupuncture and Moxibustion of China Academy of Chinese Medical Sciences (Approval No. 2013021801). Efforts were made to minimize the number and sufferings of the animals used. All sections of this study were adhered to the ARRIVE Guidelines for reporting animal research.

### Experimental design

The present study consists of three sections. The 1st section was designed to examine the effect of EA on pain thresholds of bilateral hind-paws. Thirty-six animals in this part were randomly divided into 3 groups: sham CCI control, CCI model and EA (*n* = 12 in each). The 2nd section was designed to observe different roles of astrocytes and microgliacytes in the cumulative analgesic effect of EA by using immunofluorescent staining. A total of 54 rats were randomized into 8 groups: normal control, sham CCI 6 day (D), sham CCI 18D, CCI 6D, CCI18D, sham CCI + EA2D, sham CCI + EA2 week(W), CCI + EA2D group, and CCI + EA2W, with 6 rats in each group. The 3rd section was designed to observe the effect of EA on the expression of glial fibrillary acidic protein (GFAP) of the lumbar spinal cord. Thirty rats were randomized into 5 groups: normal control, model, CCI + EA2D, CCI + EA1W and CCI + EA2W (*n* = 6 in each). Randomization scheme was created using the standard = RAND () function in Microsoft Excel.

### Establishment of chronic neuropathic pain

The chronic neuropathic pain model was established by ligature of the sciatic nerve according to Bennett’s and Xie’s report [19]. Under anesthesia (pentobarbital sodium 40 mg/kg, i.p.) and routine sterile procedures, the sciatic nerve located on the left side was exposed at the level of the middle of the thigh by blunt dissection through the biceps femoris muscle. Four constrictive ligatures (4–0 surgical suture) were tied around the sciatic nerve with about 1 mm spacing at the distal end close to the bifid site. After local application of antibiotic (sodium penicillin powder, 9000–10,000 U/rat), the incision was sutured in layers. In sham-CCI rats, the left sciatic nerve was also exposed without ligation.

### Behavioral test

The paw withdrawal latencies (PWLs, pain thresholds, PTs) of both hind paws were tested at about 9:00 AM on the day before surgery, and once again every 3 days after surgery.

### Measurement of mechanical pain threshold

Twenty minutes before testing, the rat was placed in an individual plastic enclosure compartment on the testing surface of Ugo Basile Dynamic Plantar Aesthesiometer (DPA) 37,450 (Varese, Italy) in order to allow them to habituate to the environment. Mechanical PWLs (response time, s; and force threshold, g.) of the plantar surface of hind-paws were measured by using the DPA with a Von Frey filament (0.5 mm). The measurement was repeated 3 times at an about 5 mins’ interval. In order to avoid potential tissue damage, the cutoff force was set at 50 g.

### Measurement of thermal pain threshold

The thermal PWLs were determined by using a plantar tester (Ugo Basile, 37,370). The rat was put into an animal plastic enclosure compartment on the polymethyl methacrylate pane of the plantar tester for 20 min, then a movable infra-red radiation source under the pane was focused onto the plantar surface of hind-paw to detect the PWLs. The detection for each paw was repeated 3 times, with an interval of about 5 mins between every two tests. In order to avoid potential tissue damage, the cut-off time of each heat radiation test was set at 30 s.

### Electroacupuncture treatment

After measuring the mechanical and thermal PTs on day 4 following surgery, the EA treatment was conducted. The rat was put into a self-made cloth bag with two hindlimbs exposed. Filiform needles (Suzhou Acu-moxibustion Products Factory, Suzhou, China) were swiftly inserted into bilateral Zusanli (ST36)-Yanlingquan (GB34) to a depth of about 4 mm, and respectively connected to the outputs of a HANS Apparatus (Hans-200A, Jisheng Medical Technology, Co., Ltd., Nanjing, China) for EA stimulation (1 mA, 2/15 Hz, duration of 30 min). With reference to the position of the human body, ST36 in rats is located at about 5 mm inferior to the capitulum fibulae and posterior-lateral to the hind-limb knee joint; and GB34, about 5 mm superior-lateral to ST36. The treatment was conducted once daily, continuously for 14 days. EA treatment had little adverse events except accidental minor bleeding due to needle puncturing. To avoid the adverse event, we choose thin needles of 0.3 mm and pressed the inserted acupoint after withdrawal of needles.

### Immunofluorescence labeling

At the end of each experiment, the rats were deeply anesthetized with pentobarbital sodium 40 mg/kg, i.p.. The animal was perfused transcardially with 0.9% normal saline and then 4% paraformaldehyde solution in 0.1 M phosphate buffer (PB, pH 7.4, 4 °C). The L4–5 spinal cord tissue was removed and stored in 4% paraformaldehyde solution for 2 h and subsequently in 30% sucrose solution for 48 h to prevent ice crystallization. The spinal cord tissue was sectioned on a cryostatmicrotome (Thermo, Microm International GmbH, Germany) at 40 μm for immunofluorescence staining. Free-floating tissue sections were placed in 0.01 M phosphate-buffer saline (PBS), washed with PBS Tween-20 (PBST) three times. Then the sections were blocked with 5% Normal Donkey Serum in 0.01 M PBST(135 mM NaCl, 4.7 mM KCl, 10 mM Na2HPO4, 2 mM NaH2PO4,0.5% TritonX-100) for 30 min at room temperature. They were incubated with primary antibodies (1:500) goat anti- Iba-1 (ionized calcium binding adaptor molecule 1, abcam, ab5076), 1:1000 mouse anti-GFAP (Millopore, 3670S), 1:500 mouse anti-NeuN (neuronal nuclear protein, Millopore) overnight at 4 °C. After washing with PBST three times, the sections were incubated with 1:500 Alexa Fluor 594-conjugated donkey anti-rabbit antibodies (molecular Probes, Rockford, R37119, USA)/ 1:500 Alexa Fluor 488-conjugated donkey anti-mouse (molecular Probes, Rockford, A-11055,USA) at room temperature for 2 h. For double immunofluorescence labeling of Iba-1/NeuN, the tissue sections were incubated with a mixture solution of the two primary antibodies, followed by a mixture of the two respective secondary antibodies. For the negative control, the cells were stained without primary antibodies and showed no signals. For quantification of Iba-1 and GFAP, images of the superficial DHs of lumbar spinal cord (L4–5) were observed using a fluorescence microscope (Olympus AX70, Olympus Corporation, Tokyo, Japan). The total numbers of Iba-1-positive cells in both ipsi- and contralateral DHs of 3 tissue sections of each animal were artificially counted. For determining the mean intensity of GFAP immunofluorescence of the superficial DHs using a software analysis Pro 3.1, five visual fields of each tissue section were randomly observed and analyzed.

### Western blotting analysis

The tissue samples of the dorsal part of spinal cord (L4–5, semi-sectioning along the longitudinal-horizontal direction) were removed and rapidly frozen in liquid nitrogen and stored at − 70 °C until further processing. Frozen tissues were placed into PBS containing a protease inhibitor cocktail (Roche, Mannheim, Germany), and then the target protein was extracted by protein lystate using a tissue homogenizer. The concentration of the protein was detected using the bicinchoninic acid (BCA) method with a bovine serum albumin standard. An equal amount of protein in each sample was separated by 5% or 8% sodium dodecyl sulfate polyacrylamide gel electrophoresis (SDS-PAGE), then, the resolved proteins were transferred to a polyvinylidene difluoride (PVDF) membrane (Millipore Corporation, Billerica, MA, USA). Primary antibodies used were mouse anti-GFAP(1:20,000,CST), mouse anti-β-actin (Immunoway, YM3028,1:5000) overnight at 4 °C. After washing 3 times with TBS buffer, the PVDF membranes were incubated with the secondary antibody (1:10,000, goat anti-mouse IgG, Jackson Immuno Research Laboratories, West Grove, PA, USA) for 2 h at room temperature. Immunoreactive bands were then revealed by enhanced chemiluminescence (Amersham Biosciences UK Limited, Buckinghamshire, England) using standard x-ray film (EasternKodakCo., Rochester, NY, USA). The relative intensity of the detected protein bands was analyzed with a Personal Densitometer SI (Amersham Biosciences) linked to ImageQuant 5.2 software B and the detection was carried out using an enhanced chemiluminescence(ECL) detection kit. The quantification of band intensity was carried out using an Image-Pro Plus software (Media Cybernetics, Silver Spring, MD).

### Statistical analysis

The analyzers were blinded to the EA treatment procedures while processing data and making exclusion decisions. The data of the present study were expressed as mean ± SD. The thermal and mechanical PTs were analyzed using two-way repeated measures analysis of variance(ANOVA) with one between-subject factor (EA) and one within-subject factor (time).The other data were evaluated by one-way ANOVA. Significance was determined at the level of *P* < 0.05.

## Results

### EA treatment alleviated both mechanical and thermal hyperalgesia

**Fig. 1:**
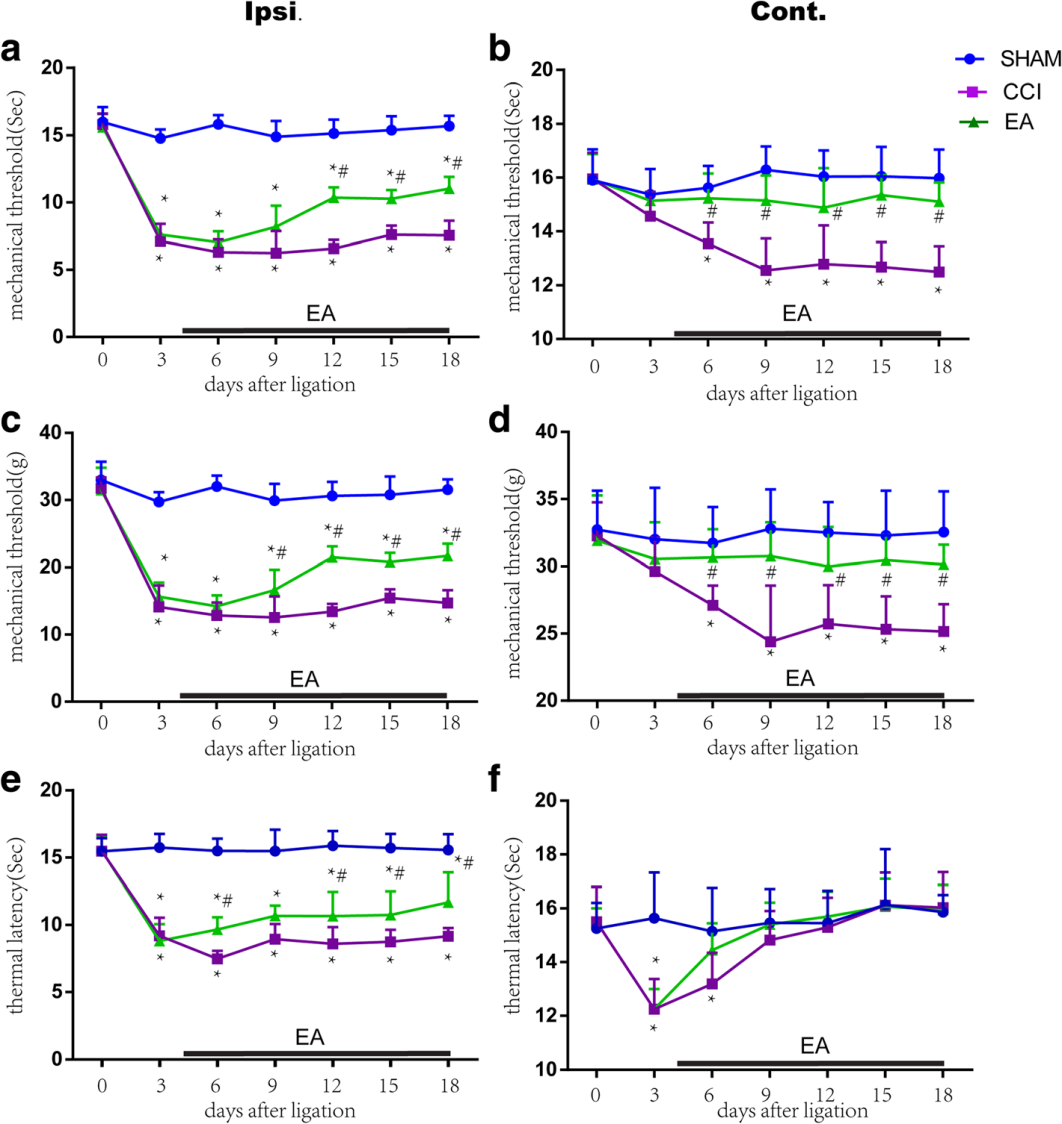
Repeated EA treatment reduced CCI-induced mechanical and thermal hyperalgesia and mirror-image pain in neuropathic pain rats (mean ± SD, *n* = 12/group). Both mechanical (response time: **a**, force threshold: **b**) and thermal paw withdrawal latencies (PWLs) at the ipsilateral (Ipsi., **a, c, e**) and contralateral (Cont., **b, d, f**) paws were observed before and every 3 days after chronic constrictive injury (CCI) of the sciatic nerve. Compared with the CCI rats, CCI-decreased mechanical PWLs were significantly increased by EA treatment from the 12th day on (time response: **a**) and 9th day on (force response: **c**) after surgery on the Ipsi. side (**a, c**), and from the 6th day on the Cont. side (**b, d**); and CCI-decreased thermal PWLs were significantly up-regulated by EA from the 6th day on after surgery on the Ipsi. side (**e**) and turned to the normal level from the 6th day on after surgery on the Cont. side (**f**), suggesting a positive effect of EA on chronic neuropathic pain and mirror-image pain. (*P < 0.05, vs the sham group; # *P* < 0.05, vs the CCI group)

The mechanical and thermal PWLs were comparable before surgery among the three groups. After CCI, the mechanical PWLs of the ipsilateral hind-paw were significantly reduced in the CCI group from day 3 on after operation, while those of the contralateral hind-paw also significantly reduced from day 6 on after surgery relevant to the sham control group (*P* < 0.05, Fig. 1a–d), suggesting an occurrence of mirror-image pain.

Following EA intervention, the PWLs of the ipsilateral paw were remarkably increased in the CCI + EA group from day 9 (force strength response) and day 12 (time response) on after surgery relevant to the CCI group (*P* < 0.05, Fig. 1a, c), while those of the contralateral paw significantly increased from day 6 on after surgery (P < 0.05, Fig. 1b, d).

After CCI, the thermal PWLs were significantly decreased at the ipsilateral hind-paw from day 3 to 18 (except day 6) and at the contralateral hind-paw on day 3 and 6 after the surgery compared with the sham operation group (P < 0.05, Fig. 1e, f).

### CCI significantly activated microgliacytes and astrocytes in lumbar spinal cord

**Fig. 2:**
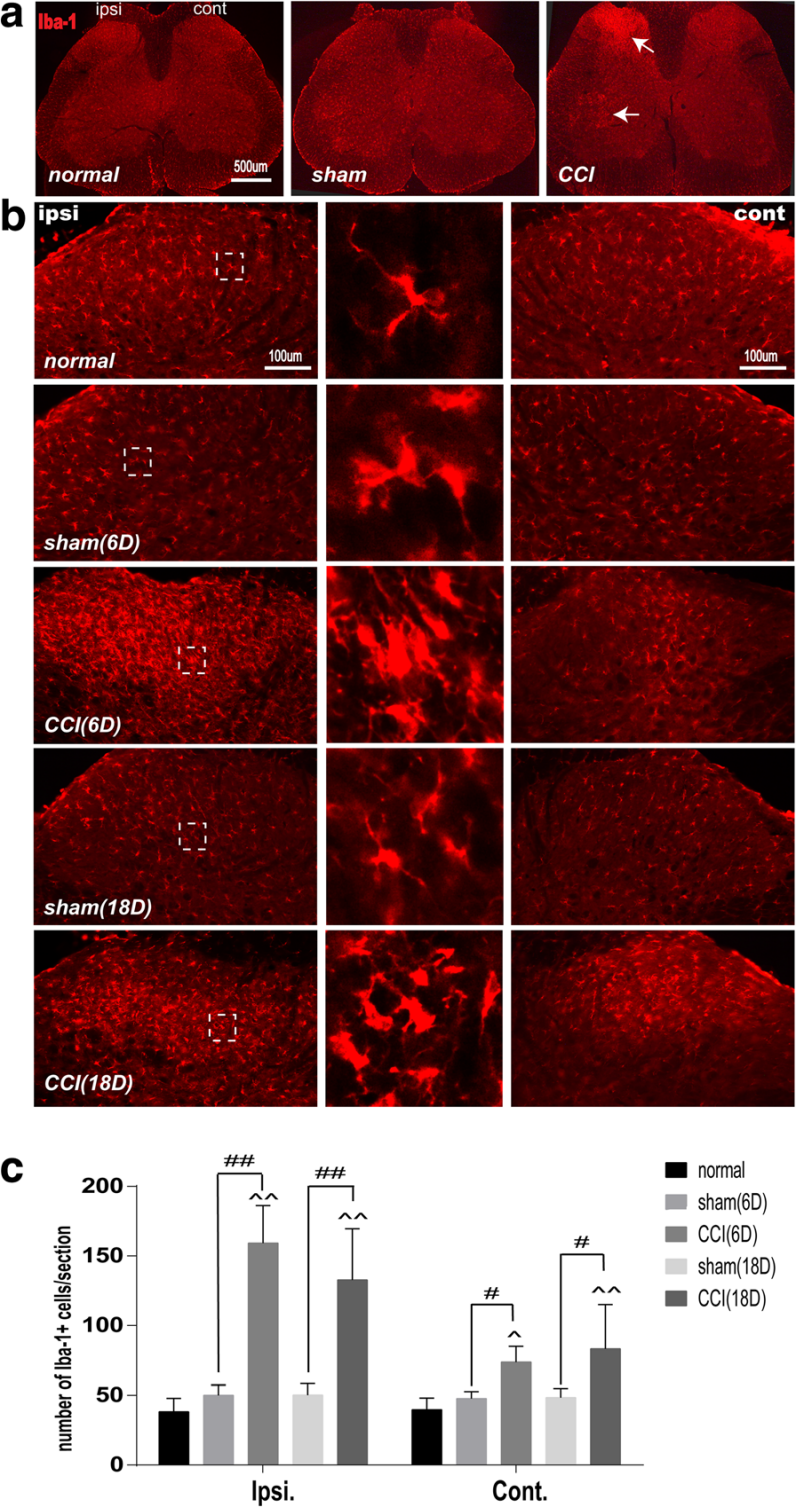
Numbers of Iba-1-labeled microgliacytes in ipsilateral and contralateral dorsal horns (DHs) of lumbar spinal cord (L4–5) on day 6 and 18 after surgery in the normal control (normal), sham operation (sham), and CCI model (CCI) groups. **a** Iba-1-labeled microglia (indicated by arrowheads) in the DHs and anterior horns of lumbar spinal cord on the ipsi. Side of CCI on day 6 after surgery. **b** An increase of numbers of the activated microgliacytes mainly distributing in the superficial layers of ipsi. and cont. DHs on day 6 (denser) and day 18 (lesser) in the 3 groups. Central column: the magnified activated microgliacytes derived from the dashed rectangular box of each picture on the left side. Ipsi: ipsilateral, Cont: contralateral. D: day. **c** Histograms showing the number of Iba-1-labeled microgliacytes in the ipsilateral and contralateral DHs of lumbar spinal cord on day 6 and 18 after sham and CCI-operation in the 3 groups (mean ± SD, *n* = 6/group). ^ *P* < 0.05, ^^ *P* < 0.01, vs the normal group; # P < 0.05, ## P < 0.01, vs the sham group

In order to determine whether the sham and CCI can activate microgliacytes and astrocytes in the DHs of lumbar spinal cord on the ipsilateral and contralateral sides of CCI, we observed changes of immunoactivity of Iba-1 (a microglial marker), and GFAP (a astrocytic marker). Under microscope it was found that on day 6 after CCI operation, the microgliacytes in the ipsilateral and contralateral DHs (laminae I-IV) and the anterior horns (lamina IX, CCI-operated side) of the lumbar spinal cord in the sham operation group had no observable changes in their shapes, presenting long processes and small cellular bodies (Fig. 2a), while those in the ipsilateral (not the contralateral) DHs of the CCI group presented ameboid shape with hypertrophic and hyperplastic bodies and fewer processes (Fig. 2b), suggesting their activation at this time-point after CCI. Additionally, the numbers of Iba-1-positive cells in the ipsilateral and the contralateral DHs were considerably increased in the CCI group compared with that of the normal and sham control groups (*P* < 0.01, *P* < 0.05,respectively).

On day 18 after CCI, the numbers of Iba-1-labeled cells in both ipsilateral and contralateral DHs were still markedly increased in the CCI group relevant to the normal (*P* < 0.01) and the sham groups (*P* < 0.05, Fig. 2c).

**Fig. 3:**
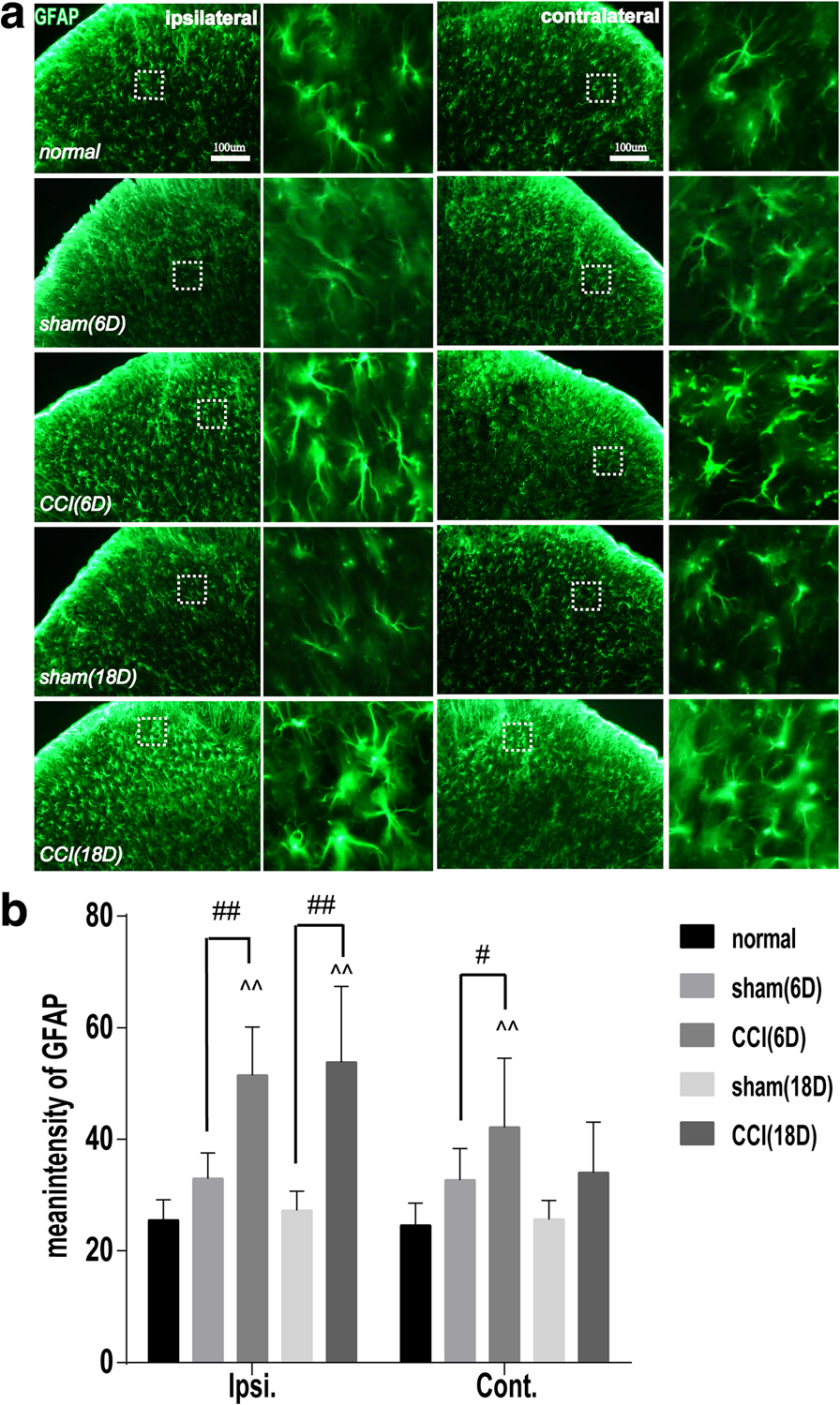
Immunofluorescent staining of glia fibrillary acidic protein (GFAP) for displaying astrocytes in Ipsi and Cont DHs of lumbar spinal cord on day 6 and 18 after CCI in rats of the five groups. **a** Representative samples of immunofluorescence staining showing an increase of the number of GFAP-labeled astrocytes in the superficial layers of DHs of lumbar spinal cord on the Ipsi and Cont sides of surgery on day 18 (relevant to day 6). In the right column of both Ipsi and Cont DH section samples, a magnification of GFAP-labeled astrocytes (got from the dashed square boxes of the tissue section samples on the left side) is shown, suggesting an increase of number of the activated astrocytes after CCI. **b** Bar graphs showing the mean immunofluorescence intensity of GFAP in the Ipsi and Cont DHs on day 6 and 18 in the 5 groups. Eight rats in each group were examined and data are expressed in Mean ± SD(n = 6/group). ^^ P < 0.01, vs the normal group; # P < 0.05, ## *P* < 0.01, vs the sham group

On day 6 and 18 after surgery, the GFAP-labeled astrocytes in both ipsilateral and contralateral DHs had thin processes and smaller cell bodies in the sham group, suggesting no activation, but exhibited an obviously hypertrophic body with thicker processes in the CCI group (Fig. 3a). The average fluorescence intensity levels of GFAP of the ipsilateral and contralateral DHs were significantly higher in the CCI 6D and CCI18D groups than in the sham 6D and sham18D groups (P < 0.01, ipsilateral side, *P* < 0.05, contralateral side, Fig. 3b).

### EA treatment inhibited CCI induced activation of microgliacytes and astrocytes

**Fig. 4:**
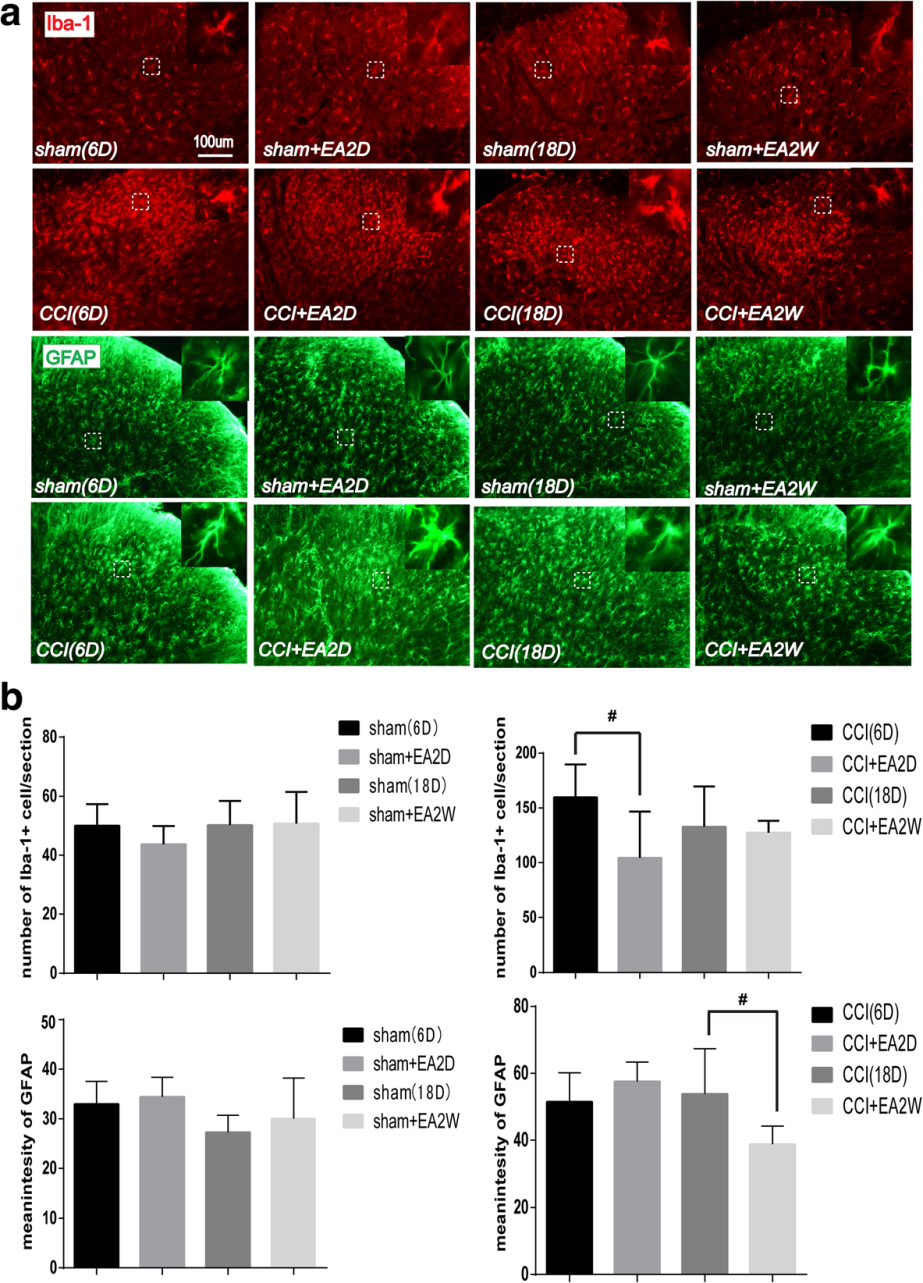
Immunofluorescence staining showing activities of microgliacytes and astrocytes in Ipsi and Cont DHs of lumbar spinal cord on day 6 and 18 after CCI in rats. **a** Representative tissue sections of immunofluorescence staining showing Iba-1-labeled microgliacytes (red) and GFAP-labeled astrocytes (green) in the Ipsi DHs of the lumbar spinal cord. In the superior-right corner of each section, the magnified microgliacyte and astrocyte from each dashed square box are shown. **b** Bar graphs showing the numbers of microgliacytes and mean fluorescence intensity values of GFAP (for astrocytes) in the Ipsi DHs on day 6 and 18 in the sham and EA groups(mean ± SD, n = 6/group). Results showed that in the early period of CCI-induced neuropathic pain, 2 days’ EA suppressed the activation of microgliacytes (not astrocytes), and in the later period, 2 weeks’ EA suppressed the activation of astrocytes. # P < 0.05, vs the CCI group)

On day 6 and 18 after surgery, no significant differences were found among the sham 6D, sham EA2D, sham 18D and sham EA18D groups in the number of Iba-1-positive cells/section and in the average fluorescence intensity of GFAP of the ipsilateral DHs of the lumbar spinal cord (*P* > 0.05, Fig. 4a, b). Whereas, after EA treatment, the number of Iba-1-positive cells/section in the ipsilateral DHs in the CCI + EA2D group (not the CCI + EA2W group) and the average fluorescence intensity of GFAP in the CCI + EA2W group (not the CCI + EA2D group) were considerably decreased (P < 0.05). It displays that both the sham operation and sham EA had no apparent influence on the activities of microgliacytes and astrocytes, but in real-CCI rats, 2 days’ EA treatment inhibited CCI-induced activation of microgliacytes and 2 weeks’ EA suppressed the activation of astrocytes in the lumbar spinal cord.

**Fig. 5:**
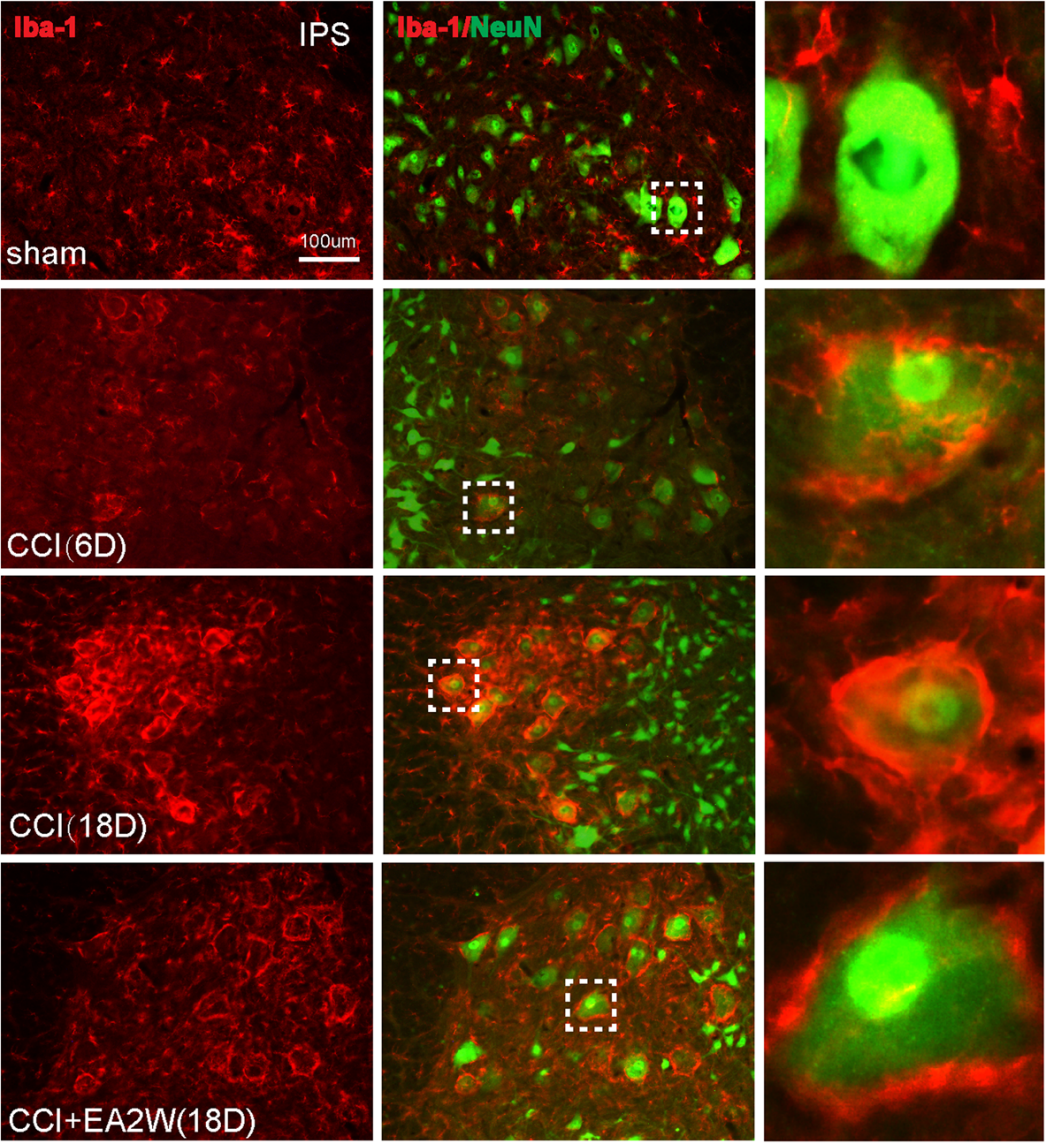
Representative section samples of dual immunofluorescent staining of Iba-1 (red) and neuronal nuclei (NeuN, green) for microgliacytes and neurons in the Ipsi ventral horns of lumbar spinal cord in the sham, CCI 6D and 18D, and CCI + EA2W groups. The images from the left to the right longitudinal rows are Iba-1-labeled microgliacytes, Iba-1 and NeuN labeled cells and magnified cells chosen from the dashed square box of each image on their individual left side. The NeuN-positive neurons were closely surrounded by many microgliacytes after CCI, suggesting a remodeling of the lumbar locomotor circuitry. NeuN, a neuronal specific nuclear protein and a biomarker for neurons

Because the sciatic nerve contains both sensory and motor fibers, the sciatic nerve injury may also damage motor neurons in the spinal cord ventral horns [20]. We thus used the immunohistochemical technique to examine the state of activities of neurons and microgliacytes in the ventral horns of lumbar spinal cord. Outcomes of immunofluorescent staining showed that in the sham group, NeuN-labeled motor neurons and Iba-1-labeled microgliacytes were separated and had no regular relationship, but on day 6 and particularly on day 18 after CCI, the NeuN-labeled motor neurons were surrounded by many activated microglial cell bodies and processes, forming a close connection with each other in the ipsilateral ventral horns (Fig. 5). Following 2 weeks’ EA intervention, the aggregation of the activated microglia around the neurons was reduced.

### EA treatment down-regulated CCI induced over-expression of GFAP

**Fig. 6:**
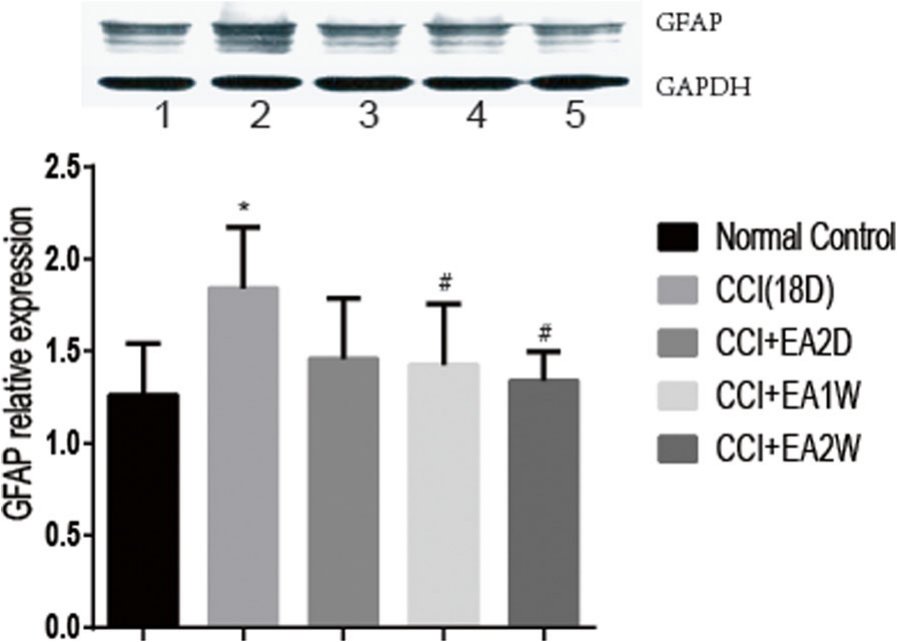
Quantitative analysis of expression levels of GFAP protein in the dorsal part of the lumbar spinal cord in rats of the five groups. Upper panel: representative western blot stripes of the 5 groups. 1: control, 2: CCI(18D), 3: CCI + EA2D, 4; CCI + EA1W, 5:CCI + EA2W. Lower panel: bar graphs showing the expression levels of GFAP protein in the 5 groups, five rats in each group were examined and the data are expressed as mean ± SD. * *P* < 0.05, vs the control group; # P < 0.05, vs the CCI group

Following CCI, the expression level of lumbar GFAP protein was significantly increased in the CCI group compared with that of the control group (*P* < 0.05, Fig. 6). After EA treatment, the expression levels of GFAP protein were significantly down-regulated in the CCI + EA1W and CCI + EA2W groups relevant to that of the CCI group (*P* < .05, Fig. 6). There was no significantly difference between the EA 1 week and EA 2 week groups in the expression level of GFAP (*P* > 0.05).

## Discussion

Chronic pain is a prevalent condition which afflicts many people and diminishes their quality of life. For relieving pain, some drugs such as opioids, non-steroidal anti-inflammatory drugs, antidepressants, etc. are commonly used, but limited in the efficacy due to adverse effects as tolerance and addiction. Increasing evidence has shown that acupuncture therapy has a positive role in relieving chronic pain, and many patients ask for acupuncture treatment [21, 22]. Our past studies demonstrated that repeated EA intervention could relieve neuropahtic pain in CCI rats, which was closely related with its effects in regulating activities of the cholinergic system and the ERK signal pathway in the hippocampus and hypothalamus [23, 24], as well as in upregulating the expression of IL-1β mRNA, TNF-α mRNA, IL-6 mRNA, and BDNF, NGF, and NT3/4 in the spinal cord [6]. In the present study, we examined the relationship between the EA analgesia and activities of glial cells in the lumbar spinal cord DHs in rats with neuropathic pain.

### Repeated EA relieves mechanical and thermal hyperalgesia

Results of the present study displayed that after CCI surgery, the mechanical (both time and force responses) and thermal pain thresholds of the ipsilateral hind-paw, and the thermal pain threshold of the contralateral hind-paw were apparently decreased from the 3rd day on and the mechanical pain threshold of the contralateral hind-paw was also remarkably decreased from the 6th day on, suggesting an induction of hyperalgesia and mirror-image pain. The mechanical hyperalgesia on the contralateral side appeared later than the ipsilateral side, but the thermal hyperalgesia on the contralateral side disappeared rapidly (from the 9th day on) after operation. It suggests that the mechanical mirror-image hypersensitivity is a persistent mode, and is different from the thermal hyperalgesia.

It was reported that EA inhibited neuropathic pain more effectively at low frequency than high frequency [25, 26], so, we employed an alternative frequency of 2 Hz/15 Hz in the present study. Although outcomes in the present study displayed that repeated EA treatment attenuated both mechanical and thermal hyperalgesia at the ipsilateral hind-paw, the effect on the thermal hyperalgesia appeared earlier (2 days) than that on the mechanical hyperalgesia (8 days in time response and 5 days in force response). EA treatment also could suppress the mechanical mirror-image pain (shown by both time and force responses) from the 6th day on after surgery, which is similar to Shen’s and colleagues’ study that strong manual acupuncture stimulation alleviated neuropathic mirror-image pain in spinal nerve ligation rats [27].

### Repeated EA suppresses glial activation

As finding a statistically significant result with 12 animals per group after EA treatment, the sample size estimation suggested that fewer animals would be required in the follow up study. Balancing this fact with the desire to reduce the use of animals, we randomly chose the rats from the behavioral testing into the next study.

Traditionally, neurons and the neural circuits are considered to be responsible for the development of chronic pain, but currently, more cumulating evidence shows an important role of glial cells including microgliacytes and astrocytes in both induction and maintenance of chronic pain [28–31]. After peripheral nerve injury, astrocytes and microglial cells are activated by many chemical substances such as substance P, calcitonin gene-related peptide (CGRP), etc., leading to hypertrophy or/and proliferation [32, 33]. The activated microgliacytes and astrocytes synthetize and release some mediators as proinflammatory cytokines, chemokines, growth factors, etc. to act on other glial cells and neurons via glial-neuronal interactions, enhancing or inducing chronic pain and mirror-image pain [29, 33–35]. The similar results were found in our experiments. When CCI-induced mechanical and thermal neuropathic pain, an activation of microglial cells (shown by increase in number and appearance of ameboid shape with hypertrophic and hyperplastic bodies and fewer processes) and astrocytes (marked by increase in immunofluorescence intensity of GFAP and occurrence of hypertrophic body with thicker processes) was found in the superficial laminae of the spinal cord DHs on the ipsilateral side of the surgery, accompanied with a closer contact between NeuN-labeled neurons and Iba-1 labeled microgliacytes in the lumbar ventral horns. On day 6 and 18 after surgery, along with the decrease of PTs, the number of microglacytes and the fluorescence intensity of GFAP were apparently increased in DHs on the contralateral side despite of being markedly lower than on the ipsilateral side.

Theoretically, the sciatic primary sensory afferent nerve fibers may take priority to carry the nociceptive information of the focus to the spinal cord DHs on the ipsilateral side and then to the higher nerve center after CCI surgery, inducing pain and protective motor reflex. It is reasonable that the neurons, microgliacytes and astrocytes were activated first in the ipsilateral DHs, followed by mirror-image pain-induced activation of glia cells in the contralateral DHs and ventral horns. Michot [36] reported that the overexpressed astrocytic metabotrophic glutamate receptor (mGluR) 5 was found mainly in the superficial laminae of the spinal cord DHs in CCI model rats. Activation of spinal glia, especially astrocytes, predominantly contributes to the development of neuropathic pain and mirror-image pain [37, 38]. In addition, some studies focusing on the relationship between glial cells and mirror-image pain demonstrated that microglial IL-1β in the spinal cord played an important role in the induction of inflammatory mirror-image pain [38]. Spinal IL-1β also inhibited astrocytic activation to mediate an induction of contralateral mechanical hyperalgesia through modulation of spinal connexin (Cx) 43 expression [39].

Accumulating evidence displays that EA intervention reduced pain by activating a variety of bioactive chemicals as opioids, serotonin and norepinephrine in the central nervous system [40]. A previous study [13] showed that the inhibition of spinal glial cell activation may contribute to the process of EA analgesia in neuropathic pain rats, and repeated EA may be useful for relieving chronic pain. In spinal cord injury (SCI) rats, it was found that the effects of acupuncture stimulation of Shuigou (GV26) and Yanglingquan (GB34) in reducing mechanical and thermal hyperalgesia were mediated in part by down-regulating GFAP expression and inhibiting JNK activation in astrocytes at L4-L5 level of the spinal cord [41]. Liang [42] also reported that EA stimulation alleviated neuropathic pain, at least in part through inhibition of spinal glial activation. These reports are basically consistent with our results of repeated EA treatment in CCI rats in the present study.

In this study, EA treatment for twice inhibited ipsilateral microglial activation on day 6 not on day 18 post-CCI, while repeated EA treatment for two weeks inhibited ipsilateral astrocytic activation on the 18th day. It suggests that the inhibition of spinal microglial activation may involve in the analgesic effect of EA in the early phase of CCI-induced neuropathic pain and the inhibition of astrocytic activation may mediate the EA analgesia in the later phase, which is similar to Liang’s and colleagues’ results [42]. These findings suggest that the ipsilateral astrocytic activation is responsible for the mechanical hyperalgesia. Following peripheral nerve injury, the activation of astrocytes is induced secondarily to the microglial response [43], however, the astrocytic activation remained longer, and was in parallel with the hypersensitivity response [44]. Moreover, astrocytic activation had a direct relationship with the elevation of mechanical allodynia [45]. It displays that the astrocytic activation induced by peripheral injuries may play a more important role in the development of chronic pain. Astrocytic inactivation also showed a close relation with EA-induced reduction of the mechanical hyperalgesia in the present study, which is basically identical to Mi’s and colleagues’ results that the analgesic effect of EA was closely related to the upregulated expression of spinal neurotrophin-3 protein and gene via inhibition of spinal GFAP (astrocytic marker) in inflammatory pain rats [46]. Moreover, repeated EA for over 1 week (not 2 days) suppressed the activation of astrocytes, which is in parallel with an increase of mechanical pain threshold, suggesting a necessary longer period of repeated EA treatment for chronic pain in clinical practice. However, the underlying mechanism remains unclear and needs to be studied in the future.

In the present study, EA treatment for twice or 2 weeks in sham CCI rats had no apparent effect on the activities of astrocytes and microgliacytes of DHs of the lumbar spinal cord on day 6 and 18 after sham operation. It suggests that the EA treatment is not a noxious stimulus for healthy animals, and the effect of EA treatment in normal animals is different from that in rats with pain conditions. Therefore, for chronic conditions, a longer period of EA intervention is necessary and has fewer side effects.

## Conclusion

Our data showed that repeated EA treatment has an analgesic effect in chronic neuropathic pain rats, which is mainly and closely related to its effect in suppressing astrocytic activation. The inhibition of astrocytic activation in the spinal cord may play a more important role than that of microglial activation in a longer EA treatment procedure for relieving chronic neuropathic pain.

## Abbreviations

BDNF: Brain-derived neurotrophic factor
CCI: Chronic constrictive injury
Cx43: Connexin 43
EA: Electroacupuncture
ERK: Extracellular signal-regulated kinase
GDNF: Glia cell line-derived neurotrophic factor
GFAP: Glia fibrillary acidic protein
IL-1β: Interleukin-1 beta
IL-6: Interleukin-6
MAPK: Mitogen-activated protein kinase
mGluR: Metabotrophic glutamate receptor
NGF: Nerve growth factor
NT3/4: Neurotrophin 3/4
PBS: Phosphate-buffer saline
PVDF: Polyvinylidene difuoride
PWL: Paw withdrawal latency
SCI: Spinal cord injury
SDS-PAGE: Sodium dodecyl sulfate polyacrylamide gel electrophoresis
TCM: Traditional Chinese Medicine
TNF-α: Tumor necrosis factor-alpha
